# Mammalian Clusterin associated protein 1 is an evolutionarily conserved protein required for ciliogenesis

**DOI:** 10.1186/2046-2530-1-20

**Published:** 2012-11-01

**Authors:** Raymond C Pasek, Nicolas F Berbari, Wesley R Lewis, Robert A Kesterson, Bradley K Yoder

**Affiliations:** 1 Department of Cell, Developmental and Integrative Biology, University of Alabama at Birmingham, 1918 University Blvd., Birmingham, AL, 35294, USA; 2 Department of Genetics, University of Alabama at Birmingham, 720 20th St. S., Birmingham, AL, 35294, USA

**Keywords:** Intraflagellar transport, Sonic hedgehog, Clusterin associated protein 1, IFT complex B

## Abstract

**Background:**

Clusterin associated protein 1 (*CLUAP1*) was initially characterized as a protein that interacts with clusterin, and whose gene is frequently upregulated in colon cancer. Although the consequences of these observations remain unclear, research of *CLUAP1* homologs in *C. elegans* and zebrafish indicates that it is needed for cilia assembly and maintenance in these models. To begin evaluating whether Cluap1 has an evolutionarily conserved role in cilia in mammalian systems and to explore the association of Cluap1 with disease pathogenesis and developmental abnormalities, we generated *Cluap1* mutant mice.

**Methods:**

*Cluap1* mutant embryos were generated and examined for gross morphological and anatomical defects using light microscopy. Reverse transcription PCR, β-galactosidase staining assays, and immunofluorescence analysis were used to determine the expression of the gene and localization of the protein *in vivo* and in cultured cell lines. We also used immunofluorescence analysis and qRT-PCR to examine defects in the Sonic hedgehog signaling pathway in mutant embryos.

**Results:**

*Cluap1* mutant embryos die in mid-gestation, indicating that it is necessary for proper development. Mutant phenotypes include a failure of embryonic turning, an enlarged pericardial sac, and defects in neural tube development. Consistent with the diverse phenotypes, *Cluap1* is widely expressed. Furthermore, the Cluap1 protein localizes to primary cilia, and mutant embryos were found to lack cilia at embryonic day 9.5. The phenotypes observed in *Cluap1* mutant mice are indicative of defects in Sonic hedgehog signaling. This was confirmed by analyzing hedgehog signaling activity in *Cluap1* mutants, which revealed that the pathway is repressed.

**Conclusions:**

These data indicate that the function of *Cluap1* is evolutionarily conserved with regard to ciliogenesis. Further, the results implicate mammalian *Cluap1* as a key regulator of hedgehog signaling and as an intraflagellar transport B complex protein. Future studies on mammalian *Cluap1* utilizing this mouse model may provide insights into the role for Cluap1 in intraflagellar transport and the association with colon cancer and cystic kidney disorders.

## Background

Cilia are complex organelles requiring hundreds of different genes for their assembly and function [1]. The assembly of the cilium is dependent on intraflagellar transport (IFT), a molecular motor-driven process that mediates the bidirectional movement of proteins between the base and tip of the cilium [2,3]. IFT was initially described in the green algae *Chlamydomonas reinhardtii* and subsequently in multiple other ciliated eukaryotes, thereby suggesting a highly conserved function.

Biochemical analysis has revealed the presence of two large distinct complexes of IFT proteins termed IFT complex A and B. Complex B is thought to mediate movement in an anterograde direction toward the tip of the cilium, while IFT complex A appears to facilitate retrograde movement to bring proteins back to the cilium base [4,5]. Each complex is necessary for proper cilia maintenance and is important for cilia-mediated signaling activities. For example, the Sonic hedgehog (Shh) pathway requires the cilium, with mutations in complex B proteins resulting in a repressed pathway, while complex A mutants have elevated signaling [6-9]. In humans, loss of ciliary function is responsible for a variety of diseases collectively referred to as ciliopathies [10]. The ciliopathies are characterized by a broad range of clinical features including neural tube defects, skeletal abnormalities, cystic kidneys, retinal degeneration, and obesity, just to name a few [11]. How loss of ciliary function contributes to this wide range of phenotypes is unknown. Therefore, the identification of novel mammalian IFT-associated genes and the generation of corresponding mutant models will provide insights into the ciliary connection to human disease and development defects.

In this regard, invertebrate model organisms have proven invaluable. One example can be seen in the case of *dyf-3*, a gene recently demonstrated to be necessary for proper ciliogenesis in the nematode worm *C. elegans*[12,13]. Subsequent studies demonstrated that a homolog of *dyf-3*, named *qilin*, is also present in zebrafish [14]. Interestingly, not only was *qilin* found to be necessary for cilia assembly and maintenance in zebrafish, but loss of function mutations in *qilin* causes a polycystic kidney disease-like phenotype similar to that observed for mutations in known IFT genes [15,16]. Although a *Chlamydomonas* homolog of DYF-3/qilin was not biochemically purified as a key component of the IFT complex, fluorescently tagged DYF-3 has been observed undergoing IFT in the cilia of *C. elegans*[17]*.* Further, mutations in *dyf-3* result in ciliary defects, indicating that the protein may be a previously unrecognized component of either the IFT B or IFT A complex [4,5,17].

There is also a human homolog of DYF-3/qilin, originally referred to as ‘hypothetical protein KIAA0643’ but later renamed clusterin associated protein 1 (CLUAP1). Cluap1 was described as a coiled-coil protein that localized to the nucleus and whose expression changed with the cell cycle. Further, *CLUAP1* was commonly upregulated in numerous colorectal carcinomas, and suppression of *CLUAP1* expression reduced the growth of colon cancer cells [18]. In addition, CLUAP1 interacts with clusterin, a protein induced by cell injury and elevated in cyst fluid in multiple cystic kidney disorders [18,19]. The cellular properties and physiological importance of CLUAP1 are unknown despite its association with the cell cycle and demonstrated alterations of CLUAP1 expression in various human disorders and diseases, as well as *in vitro* interaction with the protein clusterin [18,20].

Based on the findings in *C. elegans* and zebrafish, it was hypothesized that the mammalian homolog would have roles in IFT and cilia mediated signaling. To test this hypothesis, a *Cluap1* knockout mouse model was generated to assess the role of *Cluap1* in an *in vivo* mammalian system.

## Methods

### Generation of Cluap1 knockout allele mice

The *Cluap1* knockout allele (*Cluap1*^*tm1a(KOMP)Wtsi*^, Knockout Mouse Project Repository, Davis, CA; hereinafter referred to as *Cluap1*^*KO*^) was generated using embryonic stem cells in which a β-galactosidase-neomycin resistance fusion cassette was inserted into intron 2 of *Cluap1*. The insertion site was confirmed by genomic PCR and sequence analysis. PCR primers for genotyping were designed based on the insertion site (sequences available upon request). The embryonic stem cells containing the targeted allele were on the C57BL/6 N background and were injected into albino C57BL/6 blastocysts (C57BL/6 J-*Tyrc-2 J*; JAX Laboratories) by the UAB Transgenic Mouse Facility using standard procedures. Chimeras were then crossed with albino C57BL/6 females, and germline transmission was confirmed by the coat color of the offspring and subsequent PCR genotyping. After obtaining no homozygous mutant offspring from heterozygous matings, timed pregnancies were established to isolate embryos at the indicated gestational time point with the morning of the vaginal plug being considered embryonic day 0.5 (E0.5). Embryos were genotyped from DNA isolated from yolk sac by PCR. Mice were provided standard laboratory chow and water *ad libitum*. All procedures and studies involving mice were approved by the UAB Institutional Animal Care and Use Committee in accordance with regulations at the University of Alabama at Birmingham.

### Reverse transcription PCR analysis

RNA was isolated from *Cluap1*^*WT*^, *Cluap1*^*Het*^, and *Cluap1*^*KO*^ E9.5 embryos with Trizol reagent according to the manufacturer’s protocol (15596–026, Life Technologies, Carlsbad, CA). Once extracted, RNA was used to synthesize cDNA using the Verso cDNA kit according to the manufacturer’s protocol (AB-1453, Thermo Scientific, Pittsburgh, PA). PCR analysis was then performed using the following primers (written 5^′^ to 3^′^), which flank the sequence between the first and last exons of the *Cluap1*^*WT*^ allele: GGACTCGAGACCATGTCT and GGACCCGGGAAGAAGTCA. The following primers were also used as a positive control to confirm the presence of actin in all samples: ATGGGTCAGAAGGACTCCTA and GGTGTAAAACGCAGCTCA. All results were confirmed by repeating the experiment in at least two additional animals.

### Cluap1 antibody generation

Antisera against Cluap1 was generated in rabbits by using a 19-residue peptide (KPSRRIRKPEPLDESDNDF) starting at position 395 of the mouse protein according to the standard protocol established by Open Biosystems (Huntsville, AL, USA). Specificity of the antisera against Cluap1 was confirmed by Western blot analysis of protein extracts isolated from *Cluap1*^*WT*^, *Cluap1*^*Het*^, and *Cluap1*^*KO*^ embryos.

### Cell culture

IMCD3 cells (ATCC, Manassas, VA) were maintained in DMEM: F12 medium supplemented with 10% FBS, 1.2 g/l of sodium bicarbonate, 0.5 mM sodium pyruvate, 100 U/ml penicillin, and 100 mg/ml streptomycin. NIH3T3 cells were cultured in DMEM with 10% FBS containing 100 U/ml penicillin and 100 mg/ml streptomycin. Creation of 176-6C renal epithelial cells was derived by microdissection of the cortical collecting duct segments of the kidney as previously described by Croyle *et al.*[21]. To induce cilia formation, cells were serum starved for 24 – 48 h prior to analysis. All cells were grown at 5% CO_2_/95% air at 37°C.

### Immunoblotting

Embryonic day 9.5 embryos were isolated into ice-cold lysis buffer [137 mM NaCl, 20 mM Tris pH 8.0, 1% Triton X-100, 10% glycerol, and complete EDTA-free protease inhibitor cocktail (Roche Diagnostics, Indianapolis, IN)]. Embryos were disrupted by passage several times through a syringe attached to a 30.5-gauge needle. The lysates were incubated on ice for 30 min and vortexed every 5 min. Protein concentrations were determined by the Bradford assay (Bio-Rad Laboratories, Hercules, CA). Protein samples were resolved on a denaturing 10% Tris–HCl gel (Bio-Rad Laboratories, Hercules, CA) and transferred to an Immobilon-Psq transfer membrane (Millipore, Billerica, MA). Membranes were blocked in TBS-T (10 mM Tris–HCl, pH 7.5, 150 mM NaCl, 0.1% Tween-20) with 5% milk for 1 h and incubated with primary antibody diluted in TBS-T with 2% BSA for 16–24 h at 4°C. Membranes were probed with horseradish peroxidase (HRP)-conjugated secondary antibodies diluted in TBS-T with 1% milk for 1 h at room temperature. Secondary antibodies were detected using SuperSignal West Pico Chemiluminescent Substrate (Pierce, Waltham, MA), and bands were visualized using Blue Ultra Autorad Film (Bioexpress ISC). The following primary antibodies and dilutions were used: anti-actin (Sigma; rabbit polyclonal; 1:1,000) and anti-Cluap1 (1:1,000). The secondary antibody was HRP conjugated anti-rabbit (#31460) and was used at 1:5,000 (Pierce/Thermo Scientific, Waltham, MA).

### β-galactosidase assays

Whole kidney and heart were extracted from *Cluap1*^*WT*^ and *Cluap1*^*Het*^ mice at 8 weeks of age. Tissues were fixed overnight at 4°C in 4% PFA in PBS and subsequently washed in PBS. Tissues were then cryoprotected with 30% sucrose in PBS for 24 h and snap frozen in OCT freezing compound (Tissue-Tek, Torrance, CA). Ten-micron sections were cut with a Leica CM1900 cryostat, and sections were attached to Superfrost Plus microscope slides (12-550-15, Fisher Scientific, Pittsburgh, PA). Sections were postfixed in 4% PFA in PBS for 10 min, washed three times with lacZ wash buffer (2 mM MgCl_2_, 0.01% sodium deoxycholate, 0.02% NP-40, in 100 mM sodium phosphate buffer, pH 7.3), and then incubated in X-gal staining solution (2 mM MgCl_2_, 5 mM potassium ferrocyanide, 5 mM potassium ferricyanide, 1 mg ml^-1^ X-Gal, in PBS) at 37°C overnight. Sections were then counterstained in Fast Red for 5 min. Similarly, for whole-mount analyses E9.5 embryos and lung tissue from 8-week-old mice were fixed in 4% PFA in PBS, washed three times with lacZ wash buffer, and then incubated in X-gal staining solution at 37°C overnight.

### Immunofluorescence

Embryos and cells grown on coverslips were fixed in 4% PFA and permeabilized with 0.3% Triton X-100 in PBS with 2% donkey serum, 0.02% sodium azide, and 10 mg/ml bovine serum albumin (BSA). Embryos were then cut to make 10-μm sections. Cells and embryos were labeled with the following antibodies: anti-acetylated α-tubulin, 1:1,000 (T-6793; Sigma-Aldrich, St. Louis, MO); anti-Arl13b, 1:1,000 (a gift from Dr. Tamara Caspary, Emory University); anti-Cluap1, 1:1,000 (generated as described above); and anti_ShhN, 1:1,000 (5E1, Developmental Studies Hybridoma Bank, University of Iowa, Iowa City, IA). All incubations and washes were carried out in PBS with 2% normal donkey serum, 0.02% sodium azide, and 10 mg/ml BSA. Primary antibody incubations were performed for 16–24 h at 4°C, and secondary antibody incubations were performed for 1 h at room temperature. Secondary antibodies included Alexa Fluor-594 and 488 conjugated donkey anti-mouse and anti-rabbit (A-21203 and A-11001, Invitrogen, Carlsbad, CA). Nuclei were visualized by Hoechst nuclear stain (Invitrogen, Carlsbad, CA). Sections were mounted onto glass slides and mounted using DABCO mounting media (10 mg of DABCO (D2522; Sigma-Aldrich, St. Louis, MO) in 1 ml of PBS and 9 ml of glycerol). Slides were sealed using nail polish.

### Confocal microscopy

All fluorescence images were captured on Perkin Elmer ERS 6FE spinning disk confocal microscope, and images were processed and analyzed in Volocity version 6.1.1 software (Perkin Elmer, Shelton, CT).

### Quantitative real-time PCR analysis

Quantitative real-time (qRT) PCR analysis of RNA isolated from embryonic day 9.5 embryos was performed using iQ SYBR Green Supermix (Bio-Rad, Hercules, CA,) with the CFX96 real-time PCR detection system (Bio-Rad) as previously reported [22]. Primer pairs (from 5^′^ to 3^′^) used for qRT-PCR analysis were as follows: Patched-1: GCCAAGCCCTAAAAAAAT and ACCACAATCAATCTCCTG (previously reported by Croyle *et al.*[23]; Gli1: TCGACCTGCAAACCGTAATCC and TCCTAAAGAAGGGCTCATGGTA. The following primers for peptidylprolyl isomerase A (Ppia) were used as an internal control: CAGACGCCACTGTCGCTTT and TGTCTTTGGAACTTTGTC (both Gli and Ppia primers previously reported by Hellstrom *et al.*[24]). Samples were run in triplicate using RNA from at least three different embryos per genotype.

### Statistical analysis

The difference in gene expression between *Cluap1*^*WT*^ and *Cluap1*^*KO*^ embryos was assessed using Student’s *t*-test on log-transformed values of the relative normalized quantity of template. Significance was established at *P* < 0.01. All calculations were performed using Microsoft Excel.

## Results

### Loss of *Cluap1* is embryonically lethal

**Figure 1:**
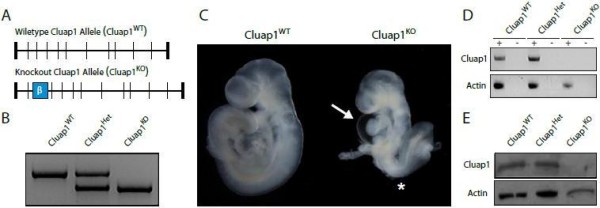
**Clusterin associated protein 1 (Cluap1) knockout mice are embryonic lethal.** (**A**) Schematic of the wild-type Cluap1 allele (Cluap1^WT^) and the Cluap1 knockout allele (Cluap1^KO^). The relative position of the β-galactosidase cassette is indicated by the blue box. (**B**) PCR genotyping of Cluap1^WT^, Cluap1^Het^, and Cluap1^KO^ embryos. (**C**) At E9.5, Cluap1^KO^ embryos are runted, have enlarged pericardial sacs (arrow), and fail to turn properly (asterisk). (**D**) RT-PCR gel showing the expression of Cluap1 transcript in both Cluap1^WT^ and Cluap1^Het^ embryos and the absence in Cluap1^KO^ embryos. Actin served as a positive template control in all samples. Reactions treated with reverse transcriptase (“+”) are alongside negative RT control samples (“- “). (**E**) Loss of the wild-type Cluap1 protein in Cluap1^KO^ embryos was determined by Western blot. Actin was used as a loading control.

Analysis of the homolog of *Cluap1* in *C. elegans* and zebrafish suggests that it is a component of the intraflagellar transport (IFT) machinery necessary for cilia assembly [17]. To assess if this role for *Cluap1* is evolutionarily conserved in mammals, a mouse embryonic stem cell line harboring a β-galactosidase cassette in intron 2 of *Cluap1* was obtained and used to generate a knockout mouse line (hereinafter referred to as *Cluap1*^*KO*^) (Figure 1A,B). We crossed Cluap1 heterozygotes (*Cluap1*^*Het*^) to produce homozygous Cluap1 knockouts (*Cluap1*^*KO*^). More than 15 different *Cluap1*^*Het*^ intercrosses producing over 150 offspring failed to yield any *Cluap1*^*KO*^ pups, indicating that loss of *Cluap1* is embryonically lethal. To determine the timing of *Cluap1* mutant lethality, we set up timed pregnancies [embryonic day 0.5 (E0.5) was the morning of copulatory plug visualization]. This revealed no surviving *Cluap1*^*KO*^ embryos between E10.5 and E18.5. However, surviving *Cluap1*^*KO*^ embryos (determined by the presence of a beating heart) were detected at E9.5. Analysis of *Cluap1*^*KO*^ embryos revealed that they were runted and exhibited enlarged pericardial sacs (Figure 1C, arrow). Most striking, however, was the failure of proper embryonic turning marked with kinks in the neural tube (Figure 1C, asterisk) when compared to wild-type siblings (*Cluap1*^*WT*^). These phenotypes are similar to those of known IFT mutants [25,26]. To determine if our *Cluap1*^*KO*^ allele was a null, we looked at both transcript and protein levels in *Cluap1*^*KO*^ embryos. Both analyses demonstrated a total loss of Cluap1 transcript and protein in the *Cluap1*^*KO*^ embryos (Figure 1D, E, Additional file 1:Figure S1).

### *Cluap1* is widely expressed in the adult and embryonic mouse

**Figure 2:**
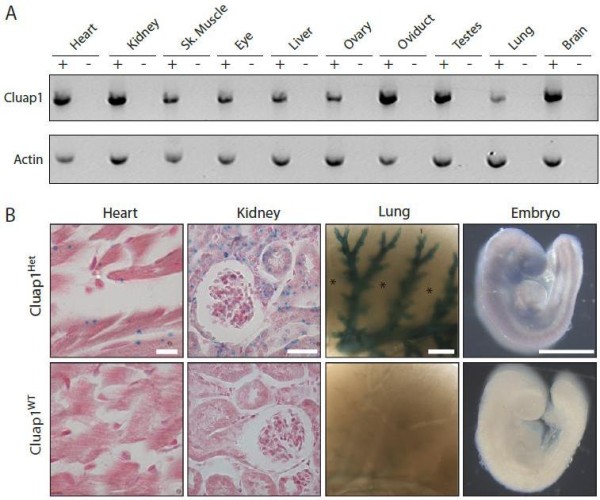
**Cluap1 is expressed in ciliated cells with a wide tissue distribution.** (**A**) RT-PCR gel showing expression of *Cluap1* in the indicated tissues; Sk. Muscle, skeletal muscle. Actin is used as a positive control. Reactions treated with reverse transcriptase (“+”) are alongside negative RT control samples (“- ”). (**B**) β-Galactosidase staining assay showing *Cluap1* expression in Cluap1^Het^ tissue in the ventricles of the heart, cortex of the kidney, lung tissue, and whole E9.5 embryo. Cluap1^WT^ control tissue samples. Heart and kidney sections were counterstained in nuclear fast red. Scale bars are 10 μm in heart sections, 30 μm in kidney sections, and 1,000 μm for whole lung tissues and embryos.

Previous studies of IFT genes have indicated they are widely expressed [27,28]. Similarly, RT-PCR analysis revealed *Cluap1* expression in all tissues tested (Figure 2A). We analyzed spatial expression of *Cluap1* using the β-galactosidase (β-gal) reporter present in the *Cluap1*^*KO*^ allele (Figure 1A). Heart, kidney, and lung tissue taken from *Cluap1*^*Het*^ mice showed β-galactosidase-positive staining (Figure 2B). The expression of *Cluap1* is markedly elevated in multiciliated cells such as the bronchioles of the lung (Figure 2B) and ependymal cells of the brain (data not show), but was absent in the alveolar parenchyma (Figure 2B, asterisks). *Cluap1* β-gal expression was also detected in cells with a single primary cilium (Figure 2B, heart and kidney).

We also stained *Cluap1*^*WT*^ and *Cluap1*^*Het*^ embryos at embryonic day 9.5, the last time point in which *Cluap1*^*KO*^ embryos are viable. In *Cluap1*^*Het*^ embryos, β-galactosidase-positive staining was present along the entire anterior-posterior axis (Figure 2B). These results show that *Cluap1* is widely expressed in ciliated tissues.

### Cluap1 localizes to the primary cilia *in vitro*

**Figure 3:**
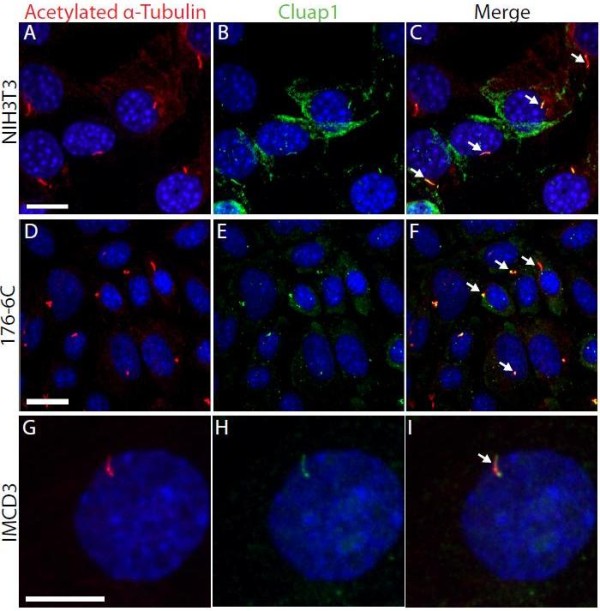
**Cluap1 localizes to primary cilia *in vitro.*** Antibody against acetylated α-tubulin (red) and Cluap1 (green) label primary cilia (arrows) in (**A-C**) NIH3T3 cells (scale bars are 14 μm). (**D-F**) 176-6C collecting duct epithelium (scale bars are 21 μm) and (**G-I**) IMCD3 cells (scale bars are 20 μm). Arrows indicate primary cilium. Nuclei are stained blue with Hoechst.

To assess Cluap1 subcellular localization, we co-immunolabled NIH3T3 cells with our Cluap1 antibody and the cilia marker acetylated α-tubulin (Figure 3A-C). Cluap1 localizes to the primary cilia and was visualized throughout the length of axoneme (Figure 3B,C). We confirmed the cilia localization in two additional independent cell lines derived from renal collecting ducts of adult mice (176-6C cells, Figure 3D-F and IMCD3 cells, Figure 3G-I).

### *Cluap1*^*KO*^ embryos lack primary cilia

**Figure 4:**
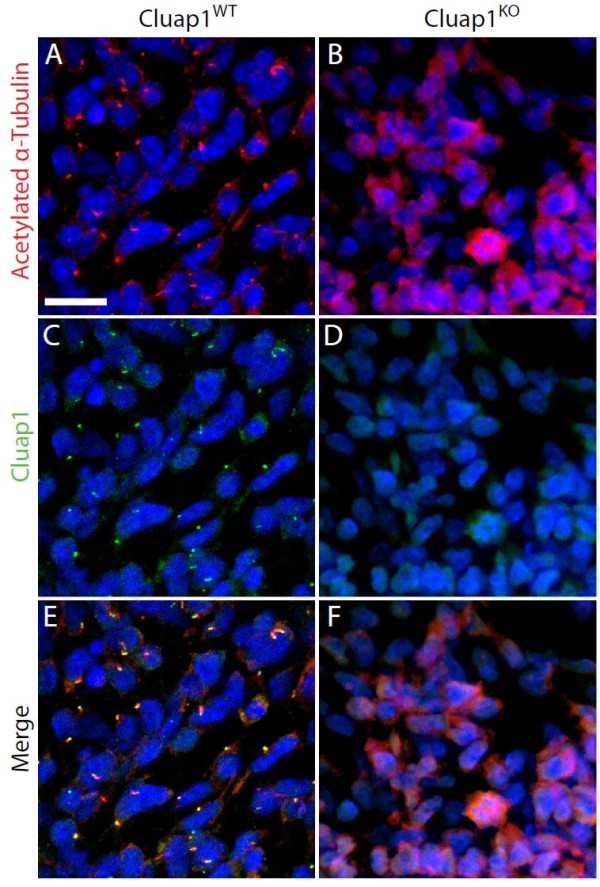
**Cluap1**^**KO**^**embryos fail to form primary cilia.** (**A,C,E**) Cluap1^WT^ E9.5 embryos were immunolabeled for the cilia marker acetylated α-tubulin (red) and Cluap1 (green) in the lateral plate mesenchyme of Cluap1^WT^ embryos. (**B,D,F**) Cluap1^KO^ embryos show a total loss of cilia in the same region. Hoechst nuclear stain in blue. Scale bar is 31.5 μm.

The improper embryonic turning and enlarged pericardial sac phenotypes seen in *Cluap1*^*KO*^ animals are similar to phenotypes observed in IFT mutants [25,26]. This finding combined with the cilia localization of Cluap1 raised the possibility that mammalian *Cluap1* is required for ciliogenesis. To test this hypothesis, E9.5 *Cluap1*^*KO*^ embryos were immunostained for the presence of cilia. Antibodies to acetylated α-tubulin showed a complete absence of cilia in sections of the lateral plate mesenchyme of *Cluap1*^*KO*^ embryos (Figure 4B,D,F), while in control *Cluap1*^*WT*^ embryos, a single primary cilium was detected on nearly every cell (Figures 4A,C,E). Thus, *Cluap1* is necessary for cilia formation in mice. Also in *Cluap1* mutant cells, the immunofluorescence showed an increase in acetylated α-tubulin staining similar to another Ift mutant [26].

### Loss of *Cluap1* disrupts Sonic hedgehog signaling

**Figure 5:**
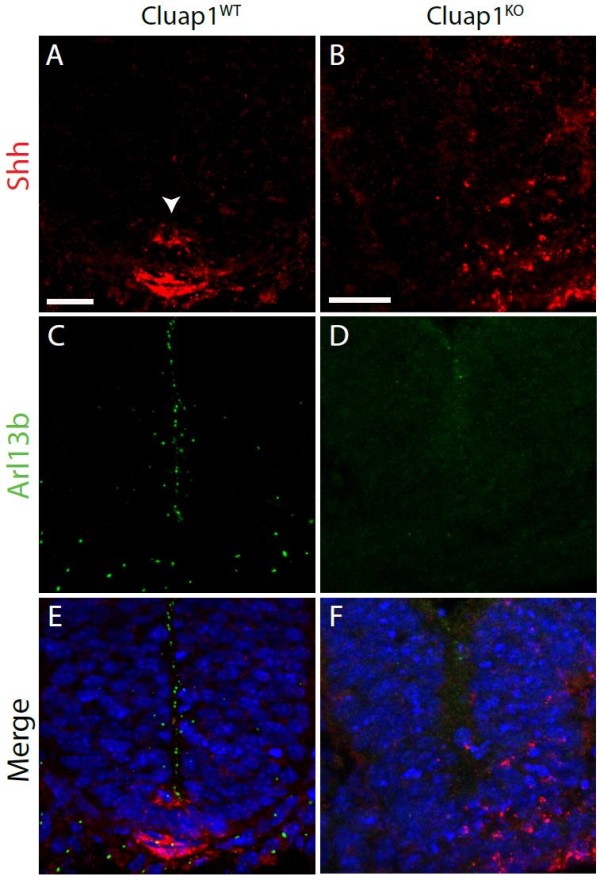
**Cluap1**^**KO**^**embryos have defects in floorplate induction.** (**A,C,E**) Cluap1^WT^ E9.5 embryos stained for Arl13b (green) show cilia in the neural tube and surrounding tissue. Staining for Sonic hedgehog ligand (red) shows a Shh immunopositive floorplate. (**B,D,F**) Cluap1^KO^ embryos show an absence of cilia as indicated by the lack of Arl13b staining. Note the lack of a clearly defined Shh immunopositive floorplate. Hoechst nuclear stain in blue. Scale bars are 21 μm.

**Figure 6:**
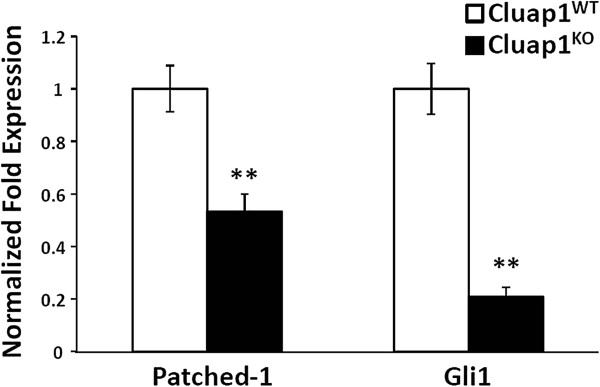
**Cluap1**^**KO**^**embryos have downregulated expression of Patched-1 and Gli1.** Real-time PCR results for the expression of *Patched-1* and *Gli1* in E9.5 Cluap1^WT^ and Cluap1^KO^ embryos demonstrate a significant decrease in expression of both *Patched-1* and *Gli1*. Expression levels are relative to control peptidylprolyl isomerase A (PPIA). Bars represent mean fold expression, and error bars are ± SEM. Asterisks represent significant difference from control (**P < 0.01, Student’s *t*-test).

Cilia are necessary for normal activation as well as repression of the Sonic hedgehog signaling (Shh) pathway, and the phenotypes in *Cluap1* mutants are consistent with defects in Hh activity [29]. To evaluate this possibility, we performed immunofluorescence analysis on the neural tubes of E9.5 *Cluap1*^*KO*^ embryos. As expected, *Cluap1*^*WT*^ embryos possessed a properly defined Shh immunopositive floorplate (Figure 5A,E arrowhead). In contrast, *Cluap1*^*KO*^ embryos stained positive for Shh ligand, but lacked a defined Shh positive floorplate (Figure 5B,F). Furthermore, staining for Arl13b, a small GTPase that localizes to primary cilia and is necessary for Shh signaling, confirmed an absence of cilia in the neural tubes of *Cluap1*^*KO*^ embryos (Figure 5D) [30,31]. To further confirm defects in Hh signaling, whole embryos were analyzed for overall Shh pathway activity by qRT-PCR analysis of *Patched-1* and *Gli1,* two downstream target genes induced by Hh. *Cluap1*^*KO*^ samples showed a significant reduction in both *Patched-1* and *Gli1* (53.3% and 20.8% of wild-type transcript levels, respectively; *p* < 0.01, Figure 6). Aside from indicating a defect in the Shh pathway, the downregulation of *Patched-1* and *Gli1* is also informative about the role of Cluap1 within the cilium itself. As previously reported, loss of function mutations in IFT complex B genes cause a downregulation of *Patched-1* and the *Gli1* transcription factors. Conversely, mutations in genes encoding IFT A complex proteins cause an increase in the *Gli1* and *Patched-1* expression [32-34]. Thus, these data indicate that *Cluap1*^*KO*^ embryos are defective in Sonic hedgehog signaling most likely because of the loss of IFT B complex function.

## Discussion

Previous data implicate homologs of *Cluap1* in cilia assembly. For example, in *C. elegans*, the *Cluap1* homolog *dyf-3* is necessary for normal cilia structure, with mutant worms failing to assemble the cilia distal segment [13]. *Dyf-3* mutant worms also display defects in cilia-regulated behaviors [12]. Similarly, in zebrafish, qilin/*Cluap1* mutant cilia degenerate in the pronephric duct, leading to subsequent cystogenesis [14,16]. Here we provide the first evidence that mammalian *Cluap1* is also a cilia protein required for cilia formation and show that mutants have characteristics consistent with Cluap1 being an IFT B complex protein.

In addition to being runted, *Cluap1*^*KO*^ mutants also failed to be properly turned by E9.5 and have an enlarged pericardial sac, indicating that cardiac insufficiency could be contributing to the midgestational lethality. Defects in embryonic turning with altered left-right axis specification along with an enlarged pericardial sac have been observed in several IFT mutant mouse models [25,26,35]. Aside from having a known role in left-right asymmetry of the heart, cilia have also been implicated in being necessary for early cardiac development through the Sonic hedgehog (Shh) signaling pathway [36,37]. Thus, it remains possible that a defect in Shh signaling during heart development could be driving the pericardial defects we observe in *Cluap1*^*KO*^ embryos.

In mice, deletion of *Cluap1* causes a total loss of cilia within the developing embryo, but this phenotype diverges slightly from studies of *Cluap1* homologs in other model organisms. An initial publication in zebrafish stated that mutants of the *Cluap1* homolog, *qilin*, were still capable of cilia assembly, leading to speculation that the protein has an accessory role in cilia maintenance or signaling [14,19]. This belief was further supported by the fact that the *Chlamydomonas* homolog of *Cluap1* was not found in biochemical analysis of IFT particles isolated from this organism’s flagella [4,5]. A follow-up report on the function of *qilin* in zebrafish did demonstrate that cilia in *qilin* mutants degenerate over time [16]. However, an independent study utilizing a morpholino approach to knockdown *qilin* revealed a more severe developmental phenotype with pronounced cilia loss [15]. This suggests maternal contribution of *qilin* mRNA in the genetic mutant is masking a role for *qilin* in early ciliogenesis. Our *Cluap1*^*KO*^ mutant mouse provides further support that this protein has an important role in ciliogenesis conserved across a diverse range of eukaryotic species.

Analysis of the *Cluap1*^*KO*^ mutant mice revealed that the Shh signaling pathway is severely disrupted. *Cluap1*^*KO*^ embryos lack a Shh-positive floorplate by E9.5 and have markedly reduced levels of *Patched-1* and *Gli1* mRNA. Significantly, mutations affecting complex A or complex B IFT proteins have different effects on the activity of the Shh pathway. IFT B gene mutations show a decrease in Shh signaling activity, while loss of IFT A genes leads to increased levels of Shh signaling [32-34]. Thus, the complete loss of cilia seen in *Cluap1*^*KO*^ mutants combined with the reduction in *Patched-1* and *Gli1* expression implies that Cluap1 is a component to the IFT B complex involved in anterograde cilia transport. However, we cannot unequivocally exclude a role for Cluap1 in ciliogenesis outside of IFT complex B.

## Conclusions

This study demonstrates a highly conserved role for mammalian *Cluap1* in cilia biology. *Cluap1* is necessary for proper mouse development, is expressed with a wide tissue distribution, and the protein localizes predominantly to the cilium axoneme. *Cluap1*^*KO*^ mutant embryos display an enlarged pericardial sac and have defects in neural tube development, possibly related to impaired Shh signaling activity. Importantly, these findings on the role of *Cluap1* in ciliogenesis and cilia-mediated signaling support the possibility of *Cluap1* being a candidate loci affected in human ciliopathy patients.

## Abbreviations

IFT: Intraflagellar transport; Cluap1: Clusterin associated protein 1; Shh: Sonic hedgehog; WT: Wild-type; Het: Heterozygous; KO: Knockout.

## Competing interests

The authors have no conflicts or competing interests to disclose.

## Authors’ contributions

RCP and NFB designed and performed experiments and wrote the manuscript. WRL performed the experiments. RAK created the mouse model. BKY designed experiments and wrote the manuscript. All authors read and approved the final manuscript.

## Supplementary Material

Additional file 1 **Figure S1.** Western blot analysis showing loss of Cluap1 protein expression in Cluap1 null embryos. A higher molecular weight nonspecific band is also detected but is not altered in Cluap1 mutant embryos. Click here for file
