# Supplementary figures and images for: Mammalian Clusterin associated protein 1 is an evolutionarily conserved protein required for ciliogenesis
Source: Cilia. 2012 Nov 1;1:20. doi: 10.1186/2046-2530-1-20 (PMC3556011; doi:10.1186/2046-2530-1-20)

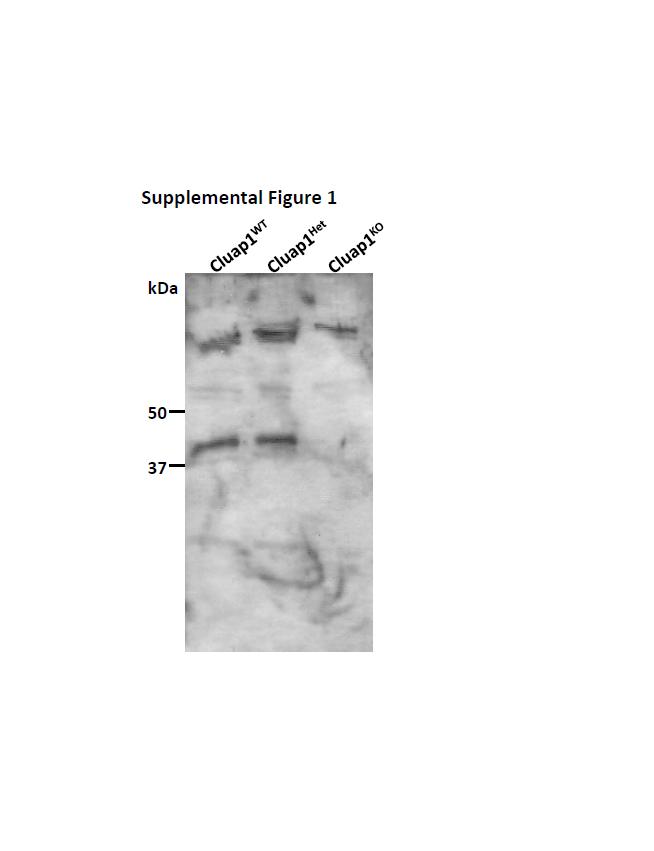

Supplement: Additional file 1 — Figure S1. Western blot analysis showing loss of Cluap1 protein expression in Cluap1 null embryos. A higher molecular weight nonspecific band is also detected but is not altered in Cluap1 mutant embryos. [file 2046-2530-1-20-S1.jpeg]
